# Can Plant Phenolic Compounds Protect the Skin from Airborne Particulate Matter?

**DOI:** 10.3390/antiox8090379

**Published:** 2019-09-06

**Authors:** Yong Chool Boo

**Affiliations:** 1 Department of Molecular Medicine, School of Medicine, Kyungpook National University, Daegu 41944, Korea; ycboo@knu.ac.kr; Tel.: +82-53-420-4946; 2 BK21 Plus KNU Biomedical Convergence Program, Kyungpook National University, Daegu 41944, Korea; 3 Cell and Matrix Research Institute, Kyungpook National University, Daegu 41944, Korea

**Keywords:** phytochemical, phenolic compounds, polyphenols, antioxidants, oxidative stress, inflammation, air pollution, particulate matter, cosmetics, skin

## Abstract

The skin is directly exposed to the polluted atmospheric environment, and skin diseases, such as atopic dermatitis and acne vulgaris, can be induced or exacerbated by airborne particulate matter (PM). PM can also promote premature skin aging with its accompanying functional and morphological changes. PM-induced skin diseases and premature skin aging are largely mediated by reactive oxygen species (ROS), and the harmful effects of PM may be ameliorated by safe and effective natural antioxidants. Experimental studies have shown that the extracts and phenolic compounds derived from many plants, such as cocoa, green tea, grape, pomegranate, and some marine algae, have antioxidant and anti-inflammatory effects on PM-exposed cells. The phenolic compounds can decrease the levels of ROS in cells and/or enhance cellular antioxidant capacity and, thereby, can attenuate PM-induced oxidative damage to nucleic acids, proteins, and lipids. They also lower the levels of cytokines, chemokines, cell adhesion molecules, prostaglandins, and matrix metalloproteinases implicated in cellular inflammatory responses to PM. Although there is still much research to be done, current studies in this field suggest that plant-derived phenolic compounds may have a protective effect on skin exposed to high levels of air pollution.

## 1. Introduction

Airborne particulate matter (PM) can damage skin cells by generating excessive reactive oxygen species (ROS) and free radicals through chemical and biological processes [1]. Alleviation or attenuation of these initial processes may help prevent the skin damage associated with air pollution [2]. As such, dermatologists and cosmetologists are engaged in uncovering safe and effective natural antioxidants to treat or prevent PM-induced skin diseases and premature aging [3].

Plant phenolic compounds, such as phenolic acids, flavonoids, stilbenoids, ellagitannins, and phlorotannins, are known to reduce the levels of cellular ROS by scavenging them directly or inhibiting their production [4]. Phenolic compounds can also enhance cells’ antioxidant capacity by stimulating the expression of enzymes involved in oxygen metabolism and xenobiotic detoxification [5,6]. Moreover, they have been shown to suppress multiple signaling pathways linked to inflammatory reactions [7,8]. 

However, whether and how plant phenolic compounds could protect the skin from PM still remains an open question to be answered with scientific evidence. Keeping this question in mind, we reviewed recent studies that report the protective effects of plant-derived phenolic compounds against oxidative stress in skin cells exposed to PM. 

## 2. PM and Its Harmful Effects

PM is a mixture of solid particles and liquid contaminants and contains inorganic and organic pollutants produced directly or indirectly from a variety of natural, artificial, and industrial sources, at sizes small enough to remain suspended in the atmosphere [9,10]. PM with a diameter of less than 10 µm (PM_10_) or less than 2.5 µm (PM_2.5_) can have major impacts on health [11,12]. PM exposure increases the incidence of inflammatory diseases in the lung, heart, blood vessels, and brain [13,14,15,16]. PM can also directly attack the eyes, causing inflammatory diseases [17].

Human skin protects the body from air pollutants, but exposure to pollutants over a prolonged or repeated period can have a serious negative impact on the skin itself [18,19,20]. Patients with imperfect skin barriers are even more susceptible to PM because of increased absorption thereof through the percutaneous tract [21,22,23]. PM can damage the skin barrier itself as well, enhancing the subsequent absorption of pollutants [24]. 

In a highly polluted atmosphere, disrupted immune function can induce or exacerbate skin diseases such as atopic dermatitis [22,25] and acne vulgaris [26,27]. A continuous inflammatory response due to PM exposure has also been associated with premature skin aging [28,29] and hyperpigmentation [30]. Simultaneous PM and ultraviolet radiation (UV) exposure exerts synergistic negative effects on the skin, associated with photo-aging and cancer [31,32]. 

## 3. Toxicity Mechanisms of PM

PM is composed of several different toxic compounds, such as transition metals and polycyclic aromatic hydrocarbons, with each constituent instigating harmful effects through different pathways and mechanisms [11,12,33]. PM can cause oxidative damage to nucleic acids, proteins, and lipids inside cells [34,35]. It also induces inflammatory reactions, disrupting skin homeostasis and barrier function [36,37]. Intracellular ROS are an inevitable byproduct of normal metabolism in oxygen-breathing organisms [38], with the mitochondrial electron transport system and oxygen-dependent metabolic enzymes as the major intracellular sources of ROS [39]. 

NADPH oxidases (NOX)-1, -2, and -4 are upregulated by PM, contributing to ROS production in cells exposed to PM [40,41,42,43]. PM-induced ROS production is intracellular calcium-dependent [44,45], and dual oxidase (DUOX)-2 which requires intracellular calcium for activation has been shown to regulate ROS production in keratinocytes exposed to PM_10_ [46] or house dust [47]. The impact of ROS on skin biology can be further increased by the Fenton reaction catalyzed by transition metals contained in PM or by the photochemical, photodynamic reactions mediated by UV in the skin [1,48]. 

PM can activate signaling pathways involving mitogen-activated protein (MAP) kinases such as extracellular signal-regulated kinase (ERK), c-Jun N-terminal kinase (JNK), and p38 MAP kinase [49,50]. PM also stimulates signaling pathways which lead to the activation of the redox-sensitive transcription factor, nuclear factor (NF)-κB [45,51]. PM has been shown to increase mRNA and protein levels of many inflammatory cytokines, including tumor necrosis factor (TNF)-α, interleukin (IL)-1α, IL-1β, IL-6, IL-8, and IL-18, in cells and in reconstructed human epidermis [50,52,53,54]. PM also induces the expression of chemokines, such as monocyte chemoattractant protein (MCP)-1, and cell adhesion molecules, such as intercellular adhesion molecule (ICAM)-1, and vascular cell adhesion molecule (VCAM)-1 [55,56]. PM has been implicated in the activation of matrix metalloproteinase (MMP)-1,-2, and -9 that are involved in the degradation of extracellular matrix components, including collagen [23,37,50,54,57].

PM has been shown to upregulate the production of prostaglandin (PG) E_2_, an eicosanoid mediator of inflammation [36,58]. In the prostaglandin synthetic pathway, arachidonic acid is converted to PGG_2_ by cyclooxygenase (COX)-1 or -2 and then spontaneously to PGH_2_ [59], and PGH_2_ conversion to PGE_2_ is catalyzed by microsomal prostaglandin E_2_ synthase (mPGES) 1 and 2 and cytosolic prostaglandin E_2_ synthase (cPGES) [60]. PM increased the expression of COX-2 by facilitating the activation of MAP kinases such as ERK, JNK, and p38 MAP kinase in keratinocytes [36,61]. Cellular mRNA levels of COX-1 and -2 and mPGES-1 and -2 have been shown to be upregulated by PM in keratinocytes [62]. 

Aryl hydrocarbon receptor (AhR) is known to regulate gene expression associated with air pollution toxicity [18,63]. AhR mediates the PM effects that increase ROS production, NOX activity, COX-2 expression, and PGE_2_ production and decreases the expression of filaggrin that physically aggregates keratin bundles and thereby enhances skin barrier function in human keratinocytes [36,64]. 

**Figure 1:**
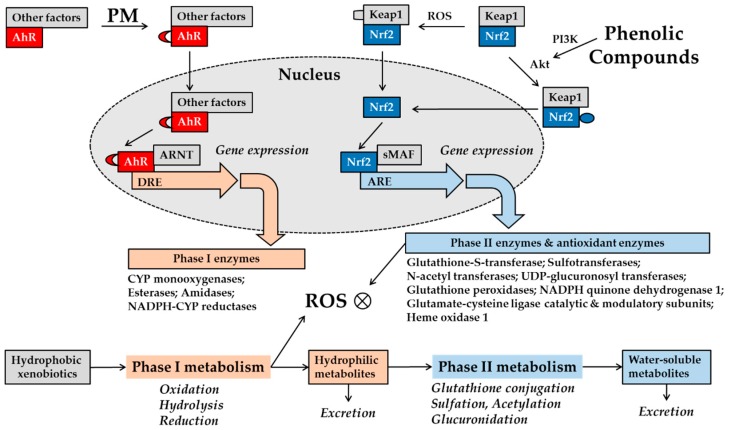
Schematic of aryl hydrocarbon receptor (AhR) and nuclear factor erythroid 2-related factor 2 (Nrf2) in metabolism phases I and II of xenobiotics. Xenobiotics, including drugs and pollutants from external sources, normally undergo phase I metabolism followed by phase II metabolism inside cells [68]. These processes render hydrophobic molecules progressively more water-soluble for excretion from the cells. Phase I metabolism oxidizes, hydrolyzes, or reduces substrates, and phase II metabolism involves conjugation reactions with glutathione, sulfate, acetate, and glucuronic acid. Organic molecules bind to AhR in a complex with protein factors in the cytosol, which promotes its nuclear translocation. In the nucleus, these factors are replaced by a homologous nuclear protein, AhR nuclear translocator (ARNT), leading to the formation of the liganded AhR–ARNT dimer, which binds to the dioxin response element (DER) on the promoter of target genes encoding phase I enzymes [69]. Nrf2 is kept at low levels, because its binding to Kelch-like ECH-associated protein 1 (Keap1) enhances ubiquitination by cullin and subsequent proteasomal degradation [67]. Under oxidative stress, certain cysteine residues of Keap1 can be modified, resulting in the release of Nrf2, which can then enter the nucleus, heterodimerize with small musculo-aponeurotic fibrosarcoma protein (sMAF), and bind to the antioxidant response element (ARE) on the promoters of target genes encoding phase II enzymes and antioxidant enzymes. Organic pollutants contained in particulate matter (PM) can stimulate AhR-dependent gene expression [18,63]. PM-induced activation of AhR can cause an excessive phase I metabolism and an increase of ROS, a condition that might be rescued by enhancing cell antioxidant capacity via an Nrf2-dependent mechanism. Many phenolic compounds derived from plants can activate Nrf2 via phosphoinositide 3-kinases (PI3K)- and Akt (protein kinase B)-dependent mechanisms, increasing downstream gene expression of antioxidant enzymes [5].

AhR in the cytosol detects xenobiotics, such as drugs and contaminants, and regulates the expression of enzymes involved in phase I metabolism of foreign substances, including cytochrome P450 (CYP)-dependent redox enzymes [65,66]. CYP enzyme activity inevitably generates ROS as byproducts, and xenobiotics themselves can generate ROS by co-opting cellular pathways such as calcium (Ca^2+^) signaling. Cells also possess a system to detect and defend their oxidative status, which involves nuclear factor erythroid 2-related factor 2 (Nrf2) [67]. The Nrf2 system mainly induces the expression of phase II metabolic enzymes, adding glutathione, glucuronic acid, and other hydrophilic molecules to the foreign substances to enhance their clearance from the cells. The system can also induce antioxidant enzymes involved in the removal of ROS and repair of oxidative damage. The AhR and Nrf2 systems are closely related to one another and thus are compared side by side in Figure 1.

## 4. Protective Effects of Plant Phenolic Compounds 

Recent studies have reported that extracts and phenolic compounds derived from different plants attenuated cellular oxidative stress induced by PM. These studies are listed in Table 1 and discussed briefly in this section.

### 4.1. Cocoa Extracts and Flavan-3-ols and Derivatives

The flavonoid group of plant phenolic compounds comprises flavonols, flavones, flavans, flavanonols, flavanones, flavan-3-ols (catechins), isoflavone, and anthocyanidins [85]. Flavonoids demonstrate antioxidant, antimicrobial, and other biological activities depending on their chemical structure, and uptake of certain flavonoids has been associated with benefits to human health [86,87]. 

Cocoa (*Theobroma cacao*) is a rich source of flavan-3-ols and procyanidins [88,89]. The cocoa products, cacao and chocolate, have been consumed by humans since ancient times, and their health benefits are supported by modern clinical evidence [90]. In 2012, Villarreal-Calderon et al. reported the effects of chocolate administration in mice housed in polluted urban air conditions [81]. Administration of chocolate was associated with a significant downregulation of cytokines such as TNF-α, IL-6, and IL-1β, and alleviation of myocardial inflammation. Chocolate was suggested to confer neuroprotective effects against air pollution in humans [43]. A human study also showed that a one-week oral administration of 4–6 g of high-flavanol cocoa per day reduced erythema formation due to UV exposure [91]. It has been suggested that cocoa-derived phenolic compounds can have positive effects on skin health [92], but their effects on skin exposed to air pollution have yet to be directly addressed. 

### 4.2. Green Tea Extracts and Catechins with Galloyl Residues

Green tea (*Camellia sinensis*) is an easily accessible source of dietary antioxidants thought to positively impact human health and help prevent disease [4]. Green tea is a rich source of catechins with galloyl residues [93], and green tea extract is frequently used as an active ingredient in cosmetics [94,95].

In a recent study by Seok et al., (−)-epigallocatechin gallate (EGCG) rescued the viability of human epidermal keratinocytes exposed to PM [75]. It also lowered ROS and mRNA expression of NOX-1, NOX-2, TNF-α, IL-1β, IL-6, IL-8, and MMP-1 stimulated by PM. The protein levels of TNF-α, IL-1β, IL-6, and IL-8 were increased by PM, and these changes were mitigated by (−)-EGCG. In another study by Wang et al., (−)-EGCG decreased intracellular ROS and increased cell viability in dermal fibroblasts exposed to PM [76]. (−)-EGCG also restored collagen synthesis while decreasing the expression of MMPs via NF-κB, activator protein 1 (AP-1) and MAP kinase signaling pathways. These results suggest that (−)-EGCG may attenuate the oxidative stress and inflammatory responses of these cells caused by PM.

### 4.3. Other Flavonoid-Rich Plant Extracts and Other Flavonoids

*Camellia japonica* leaf extracts contain phenolic compounds such as quercetin, rutin, and other flavonoids [96,97]. *C. japonica* flower extract directly scavenges ROS in vitro and induces cellular antioxidant enzymes such as superoxide dismutase, catalase, and glutathione peroxidase in human keratinocyte HaCaT cells [98]. *C. japonica* flower extract inhibited urban dust-induced ROS generation, AhR activation, and MMP-1 expression in human dermal fibroblasts [84]. In an ex vivo test on human skin explants, urban dust increased the levels of lipid peroxidation and MMP-1 expression and decreased the levels of collagen; however, these changes were prevented by pretreatment of the tissue with *C. japonica* flower extract [84].

*Astragalus mongholicus* extract and its component, formononetin, rescued the expression of keratins that had been impaired by PM exposure in HaCaT keratinocytes and in a 3D reconstructed human skin model [80]. PM increased the levels of p53, Bcl2-associated X protein (Bax), cleaved caspase-3 and poly (ADP-ribose) polymerase (PARP), all involved in apoptotic cell pathways; these changes were ameliorated by *A. mongholicus* extract and formononetin, suggesting protective effects against polluting agents.

Eupafolin, a flavone found in *Phyla nodiflora*, has been shown to inhibit COX-2 expression and PGE_2_ production in HaCaT cells exposed to PM [70]. It also suppressed PM-induced intracellular generation of ROS, activation of NOX, activation of MAP kinases such as ERK, JNK, and p38, and activation NF-κB. Topical treatment with eupafolin lowered COX-2 expression in vivo in the epidermal keratinocytes of PM-treated mice. Eupafolin nanoparticles, prepared by precipitation using the nanoparticle carriers Eudragit E100 and polyvinyl alcohol, showed enhanced aqueous solubility and skin penetration compared with free eupafolin. Eupafolin nanoparticles also showed superior antioxidant and anti-inflammatory properties than those of free eupafolin in HaCaT cells [71].

Afzelin is a flavonol glycoside (kaempferol 3-*O*-rhamnoside) found in *Thesium chinense* that inhibited mRNA expression and protein secretion of IL-1α, but not IL-1β and IL-6 in HaCaT cells exposed to PM [79]. It suppressed PM‑induced intracellular ROS generation and activation of p38 MAP kinase and of the transcription factor AP‑1 components c‑Fos and c‑Jun.

### 4.4. Grape Extracts and Resveratrol

Grapes (*Vitis vinifera*) are an easily accessible source of bioactive polyphenols including stilbenoids and proanthocyanidins [89,99,100,101]. Extracts derived from grape seeds and other grape components have shown favorable effects on human skin health and beauty [102,103]. Resveratrol, a stilbenoid compound contained in grapes, has been shown to have antioxidant, antimelanogenic, anti-inflammatory, antimicrobial, anticancer, and cardioprotective effects [104,105].

Resveratrol antioxidant and anti-inflammatory activities have been shown in human fibroblast-like synoviocytes exposed to PM [73]. Resveratrol reduced PM-induced COX-2 expression and PGE_2_ production, the upregulation thereof having been implicated in many inflammatory diseases. Resveratrol also reduced PM-induced NOX activity, ROS generation, and phosphorylation of Akt, ERK1/2, and p38 MAP kinase and attenuated PM-enhanced NF-κB p65 phosphorylation and NF-κB promoter activity. These results suggest that resveratrol may help combat PM-induced synovial joint inflammation in rheumatoid arthritis.

Choi et al. showed that resveratrol restored survival rates in human epidermal keratinocytes exposed to PM [74]. Resveratrol also alleviated PM_10_-induced ROS production and IL-6 expression at the mRNA and protein levels. Of interest, resveratrol had no effect on the mRNA levels of TNF-α or IL-8, and even enhanced IL-1β mRNA in PM_10_-exposed cells. This seemingly peculiar property of resveratrol occurs because the compound can increase or decrease inflammatory reactions and inhibit or promote apoptosis, depending on the cell type and microenvironment [106,107,108,109].

### 4.5. Pomegranate Extracts and Ellagitannins

Pomegranate (*Punica granatum*) is rich in polyphenols, the major components thereof being ellagitannins such as ellagic acid and punicalagin (a mixture of α and β anomers) [110,111], which have potent free-radical scavenging properties [112].

In a study by Park et al., pomegranate peel extract lowered the levels of ROS production and the expression of inflammatory cytokines such as TNF-α, IL-1β, and MCP-1 in PM-exposed THP-1 human acute monocytic leukemia cells [55]. The extract also reduced the expression of ICAM-1, but not VCAM-1, in monocytes and attenuated the adhesion of monocytes to endothelial cells in vitro [55]. Punicalagin and ellagic acid showed comparably attenuated PM-induced monocyte adhesion to endothelial cells, but the latter showed significant cytotoxicity.

In a subsequent study, punicalagin rescued the viability of PM-exposed human epidermal keratinocytes and attenuated ROS production therein [75]. It also suppressed PM-induced expression of NOX-1 and NOX-2 involved in ROS production and of TNF-α, IL-1β, IL-6, IL-8, and MMP-1 involved in inflammatory reactions. These results support the anti-inflammatory activity of punicalagin previously demonstrated in different experimental conditions [113,114,115].

### 4.6. Marine Algae Extracts and Phlorotannins

Marine algae have emerged as a relatively new resource for bioactive cosmetic ingredients [116,117]. Brown algae are unique in containing phlorotannins, such as eckol and dieckol, produced by the polymerization of phloroglucinol [118].

*Ecklonia cava* is a perennial brown macroalga and is found in the coastal area of East Asia [119]. It is a rich source of phlorotannins with a range of bioactivities [120]. The total phenolic content of *E. cava* extract is relatively high compared to that of many other marine algae [77]. *E. cava* extract and its constituents, eckol and dieckol, attenuated cellular lipid peroxidation in HaCaT keratinocytes exposed to PM [77]. Eckol and dieckol also downregulated the mRNA and protein levels of inflammatory cytokines, such as TNF-α, IL-1β, IL-6, and IL-8, in human epidermal keratinocytes exposed to PM [77].

*E. cava* extract attenuated PGE_2_ production in HaCaT cells exposed to varying concentrations of PM_10_ [62]. Dieckol purified from *E. cava* extract also exhibited similar effects. Both *E. cava* extract and dieckol suppressed the expression of the *COX-1*, *COX-2*, *mPGES-1*, and *mPGES-2* genes involved in PGE_2_ synthesis. In a 3D reconstructed skin model, dieckol also attenuated the morphological changes induced by PM.

In a study by Zhen et al., eckol has been shown to decrease the levels of ROS and inhibit oxidative damage to lipids, proteins, and DNA in PM-exposed HaCaT keratinocytes [78]. Eckol has been shown to attenuate PM-induced cell apoptosis by modulating MAP kinase signaling pathways [78].

Diphlorethohydroxycarmalol is a phlorotannin found in the brown alga *Ishige okamurae* [121] and has been shown to attenuate ROS formation, oxidative modifications to DNA, lipids, and proteins, endoplasmic reticulum (ER) stress, autophagy, and apoptosis induced by PM in human HaCaT keratinocytes [83]. It also attenuated oxidative damage and inflammatory reactions in HR-1 mice [83].

## 5. Mechanisms of Action of Plant Phenolic Compounds

Phenolic compounds in the plant kingdom demonstrate many different chemical structures but often have common chemical and biological activities. Some differences do, however, depend on the structure. A common characteristic is the presentation of phenolic hydroxyl groups that easily donate electrons and confer antioxidant activity. Phenolic compounds can scavenge different kinds of ROS or free radicals and, after these reactions, they become relatively stable radicals that react among themselves, terminating oxidative chain reactions. Many phenolic compounds activate signal transduction systems, such as PI3K- and Akt-dependent pathways that enhance cell survival mechanisms. In this case, the transcription factor Nrf2 is activated through phosphorylation, and the expression of downstream genes increases; as a result, the cell’s antioxidant capacity and resistance to oxidative stress are increased.

Table 2 summarizes studies providing an experimental basis for the chemical and biological properties of the various phenolic compounds. Based on the evidence, plant-derived phenolic compounds are assumed to confer antioxidant and anti-inflammatory activities via multiple mechanisms, including direct scavenging of ROS or reducing ROS generation in cells and enhancing cellular antioxidant capacity via Nrf2 activation. Through these direct and indirect antioxidant actions, the phenolic compounds can suppress the activation of redox-sensitive signaling pathways which lead to the gene expression of cytokines, chemokines, cell adhesion molecules, prostaglandins, and matrix metalloproteinases involved in the inflammatory response of cells exposed to PM.

Phenolic compounds can inhibit the catalytic activity of protein kinases involved in MAP kinase and NF-κB pathways [141]. In addition, they can inhibit the catalytic activity of metabolic enzymes involved in prostaglandin synthesis [142,143]. The catalytic activities of NOXs and MMPs can also be inhibited by certain phenolic compounds [144,145,146]. Therefore, it is considered that certain phenolic compounds can impart anti-inflammatory activity in cells exposed to PM through direct inhibition of target enzyme reactions, in addition to the other mechanisms mentioned above.

It has been reported that several phenolic compounds modulate AhR activity in different directions depending on the individual phenolic compounds tested and the specific experimental conditions, resulting in the increase or decrease of downstream gene expression [147,148,149,150,151]. Since the activation of AhR is a necessary process for the removal of toxic substances, suppressing this process may not necessarily be beneficial for cells. Nonetheless, either activation or inhibition of AhR-mediated gene expression is not considered a common mechanism of action of phenolic compounds, limiting its utility as a strategy for reducing excessive oxidative stress due to PM in cells.

## 6. Clinical Trials

PM toxicity to the skin has been evaluated indirectly by comparing the skin of residents from two regions with different levels of air pollution [152,153]. No standardized method has been established for validating the antipollution efficacy of cosmetic products on human skin. A study reported the protective efficacy of antioxidant-containing cosmetics on human skin exposed to air pollution [154]. Milani et al. tested a cosmetic serum containing *Deschampsia antartica* extract, ferulic acid, and vitamin C on the face of 20 women living in a high-pollution area in Rome [154]. The study was conducted over four weeks, and the results showed the product significantly improved skin barrier function as determined by transepidermal water loss (−19%) and enhanced skin brightness as determined by individual typology angles in color (+7%). The product also reduced oxidative stress as determined by squalene peroxide/squalene ratio (−16%) in sebum samples taken from the forehead with a swabbing method.

Due to ethical issues, the direct application of toxic air pollutants to human skin is not permitted, and few studies have applied these types of methods to evaluate the efficacy of cosmetics [155,156]. Giacomelli et al. evaluated the human skin protective effects of a multicomponent powder consisting of grape seed, green tea, and oak wood extracts, against metal deposition in a polluted environment [156]. Each hemi-face of 30 healthy volunteers was treated with a cream formulation of the multicomponent powder or a placebo cream. Following a six-hour exposure in a polluted area, stratum corneum samples of face skin were obtained using the tape-stripping method and analyzed for heavy metal content. There was a significant increase over base line in the metal content of the skin on the hemi-faces treated with the placebo cream, and these changes were not observed on the hemi-faces treated with the multicomponent powder.

Although these studies are promising, there remains a need for the development of standardized human testing methods to yield data that are objective, universal, and comparable to verify the antipollution effects of products used on human skin. Skin protection with plant-derived extracts and phenolic compounds must also be validated through standardized human testing.

## 7. Conclusions and Future Directions

**Figure 2:**
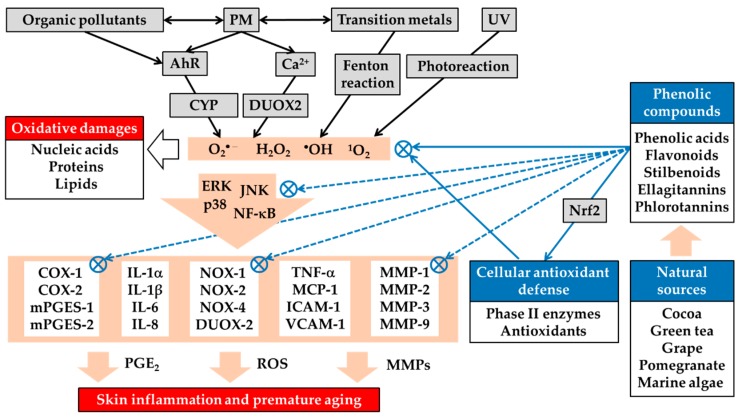
Airborne PM-induced oxidative stress in the skin and its attenuation by plant-derived phenolic antioxidants. PM stimulates the generation of ROS, such as superoxide anion radical (O_2_•^−^), hydrogen peroxide (H_2_O_2_), hydroxyl radical (•OH), and singlet oxygen (^1^O_2_), by mechanisms that include the AhR/cytochrome P-450 (CYP) pathway, calcium (Ca^2+^), and dual oxidase 2 (DUOX2)-dependent mechanisms, as well as Fenton reaction catalyzed by transition metals and photochemical or photodynamic reactions resulting from ultraviolet radiation (UV). PM-induced ROS can cause oxidative damage to nucleic acids, proteins, and lipids and activate multiple signaling pathways, such as the extracellular signal-regulated kinase (ERK), c-Jun N-terminal kinase (JNK), p38 mitogen-activated protein (MAP) kinase, and nuclear factor (NF)-κB, linked to gene expression of inflammatory mediators, such as cytokines, chemokines, cell adhesion molecules, and matrix metalloproteinases (MMPs), and synthesis of prostaglandin (PG) E_2_. Many plants, such as cocoa, green tea, grape, pomegranate, and some marine algae, are rich sources of phenolic acids, flavonoids, stilbenoids, ellagitannins, phlorotannins, and other phenolic compounds. These phenolic compounds can directly scavenge ROS from various sources and can enhance the cellular antioxidant capacity by Nrf2-dependent mechanisms. Through these direct and indirect antioxidant activities, the phenolic compounds can suppress the redox-sensitive signaling pathways leading to the gene expression of inflammatory mediators. Phenolic compounds can also directly inhibit the catalytic activity of protein kinases in the signaling pathways and other metabolic enzymes involved in ROS generation, prostaglandin synthesis, and extracellular matrix degradation. Plant-derived phenolic antioxidants can thereby alleviate oxidative stress and inflammatory skin diseases, such as atopic dermatitis and acne vulgaris, and premature skin aging caused by PM. Some phenolic compounds can increase or decrease AhR-dependent downstream gene expression, and can have positive or negative effects on skin health. NOX, NADPH oxidase; COX, cyclooxygenase; mPGES, microsomal prostaglandin E_2_ synthase; IL, interleukin; TNF, tumor necrosis factor; MCP, monocyte chemoattractant protein; ICAM, intercellular adhesion molecule; VCAM, vascular cell adhesion molecule.

Recent studies have revealed at least some of the molecular mechanisms that underlie the negative effects of PM on the skin and suggest that oxidative stress and the expression of inflammatory mediators are at the center of this phenomenon. It has also been suggested that the physicochemical and biological processes associated with the adverse effects of PM could be inhibited by plant-derived phenolic compounds with antioxidant and anti-inflammatory effects, by the direct scavenging of ROS or the enhancement of cellular antioxidant capacity via Nrf2 activation. These compounds could be promising active ingredients to address the air pollution that exacerbates skin diseases and promotes premature skin aging, as illustrated in Figure 2. To date, however, most studies have been limited to laboratory experiments using cultured epidermal keratinocytes or dermal fibroblasts, and direct clinical evidence awaits future studies.

The ideal approach for the prevention and alleviation of the adverse effects of PM on the skin is to reduce exposure to air pollution. Experts recommend that outdoor activity be minimized in highly polluted areas and conditions and that individuals protect their skin and body by wearing protective clothing. Dermatological and cosmetological approaches are a secondary approach to attenuate PM-induced deleterious effects on the skin. The composition of PM varies depending on collection place and time but commonly includes inorganic substances such as heavy metals and organic substances such as polycyclic aromatic compounds. It is important to find the right type and concentration of phenolic compounds to protect the skin under different conditions, depending on the composition and concentration of specific harmful PM. It must also be remembered that certain phenolic compounds have prooxidant side effects depending on the circumstances [157].

It is believed that various natural antioxidants such as phenolic compounds, carotenoids, and vitamins can help prevent skin inflammation, premature aging, and pigmentation disorders [105,158,159,160]. The clinical efficacy of the antioxidants will depend not only on their activity potency but also on the reach of their action targets [161,162]. Therefore, it is important to select potent antioxidants that have high skin absorption and target reachability. In addition, there is a need for the development of techniques to enhance the transport and efficacy of selected compounds using advanced formulation techniques such as liposomes, microemulsions, and nanoparticles [71,163,164,165].

Although there remains much research to be done, current studies in this field have produced a consensus that vegetable polyphenols are likely to have positive effects on the skin exposed to air pollution. In the future, it is hoped that this research will be expanded and direct evidence pursued to select plant-derived extracts and phenol compounds that can help protect human skin health and beauty.

## Figures and Tables

**Table 1:** Protective effects of plant extracts and phenolic compounds against oxidative stress and inflammation induced by airborne PM.

Studies	Models	Targets	Materials	Literature
In vitro	HaCaT keratinocytes	Skin inflammation	Eupafolin from *Phyla nodiflora*	Lee et al., 2016 [70]
	HaCaT keratinocytes	Skin inflammation	Eupafolin nanoparticles	Lin et al., 2016 [71]
	HaCaT keratinocytes	Skin inflammation	Nanoparticles containing 7,3′,4′-trihydroxyisoflavone	Huang et al., 2018 [72]
	Fibroblast-like synoviocytes	Rheumatoid arthritis	Resveratrol	Tsai et al., 2017 [73]
	HaCaT keratinocytes, 3D-skin models	Skin inflammation	Resveratrol, Resveratryl triacetate	Choi et al., 2018 [74]
	Monocytic THP-1 cells, EA.hy926 endothelial cells	Inflammation	*Punica granatum* extract, Ellagic acid, Punicalagin	Park et al., 2016 [55]
	Human epidermal Keratinocytes	Skin inflammation	Punicalagin, (−)-Epigallocatechin gallate.	Seok et al., 2018 [75]
	Human dermal fibroblasts	Skin inflammation	(−)-Epigallocatechin gallate	Wang et al., 2019 [76]
	HaCaT keratinocytes	Skin inflammation	*Ecklonia cava* extract, Eckol, Dieckol	Lee et al., 2018 [77].
	HaCaT keratinocytes, 3D-skin models	Skin inflammation	*E. cava* extract, Dieckol	Ha et al., 2019 [62]
	HaCaT keratinocytes	Skin inflammation	Eckol	Zhen et al., 2019 [78]
	HaCaT keratinocytes	Skin inflammation	Afzelin from *Thesium chinense*	Kim et al., 2019 [79]
	HaCaT keratinocytes, 3D-skin models	Skin inflammation	Formononetin from *Astragalus mongholicus.*	Nguyen et al., 2019 [80]
In vivo	Mice	Cardiac inflammation	Chocolate	Villarreal-Calderon et al., 2012 [81]
	Rats	Bronchial inflammation	*Eucheuma cottonii* extract	Saputri et al., 2014 [82]
	HaCaT keratinocytes, HR-1 hairless mice	Skin inflammation	Diphlorethohydroxycarmalol from *Ishige okamurae*	Zhen et al., 2019 [83]
Ex vivo	HaCaT keratinocytes, Human skin explants	Skin inflammation	*Camellia japonica* extract	Kim et al., 2019 [84]

**Table 2:** Potential mechanisms of antioxidant action for plant-derived phenolic compounds.

Phenolic Compounds	(1) Scavenges ROS or Reduces Their Generation	(2) Enhances Cellular Antioxidant Capacity by Activating Nrf2
Procyanidins	Saint-Cricq De Gaulejac et al., 1999 [122]	Rodriguez-Ramiro et al., 2012 [123] Song et al., 2019 [124]
(−)-EGCG	An et al., 2014 [125]	Na et al., 2008 [126]
Resveratrol	Mahal et al., 2006 [127] Holthoff et al., 2010 [128]	Kim et al., 2019 [129]
Quercetin	Aherne et al., 2000 [130] Lopez-Lopez et al., 2004 [131]	Schadich et al., 2016 [132]
Rutin	Aherne et al., 2000 [130]	Sthijns et al., 2017 [133]
Punicalagin	Kulkarni et al., 2007 [112]	Xu et al., 2015 [134]
Ellagic acid	Priyadarsini et al., 2002 [135]	Ding et al., 2014 [136]
Dieckol	Cui et al., 2019 [137]	Lee et al., 2015 [138]
Eckol	Kang et al., 2005 [139]	Kim et al., 2010 [140]

Abbreviations: ROS, reactive oxygen species; Nrf2, nuclear factor erythroid 2-related factor 2; EGCG, epigallocatechin gallate.
